# Coprecipitation as a One-Step Se Separation for Determination of Isotope Ratios Completed with Revised Uncertainty Evaluation

**DOI:** 10.1021/acs.analchem.3c04210

**Published:** 2024-02-19

**Authors:** Jakub Karasiński, Klaudia Tetfejer, Piotr Radziński, Andrii Tupys, Anna Gambin, Ewa Bulska, Ludwik Halicz

**Affiliations:** † Faculty of Chemistry, Biological and Chemical Research Centre, University of Warsaw, Żwirki i Wigury 101, Warsaw 02-093, Poland; ‡ Institute of Informatics, University of Warsaw, Stefana Banacha 2, Warsaw 02-097, Poland; § Geological Survey of Israel, Jerusalem 9692100, Israel

## Abstract

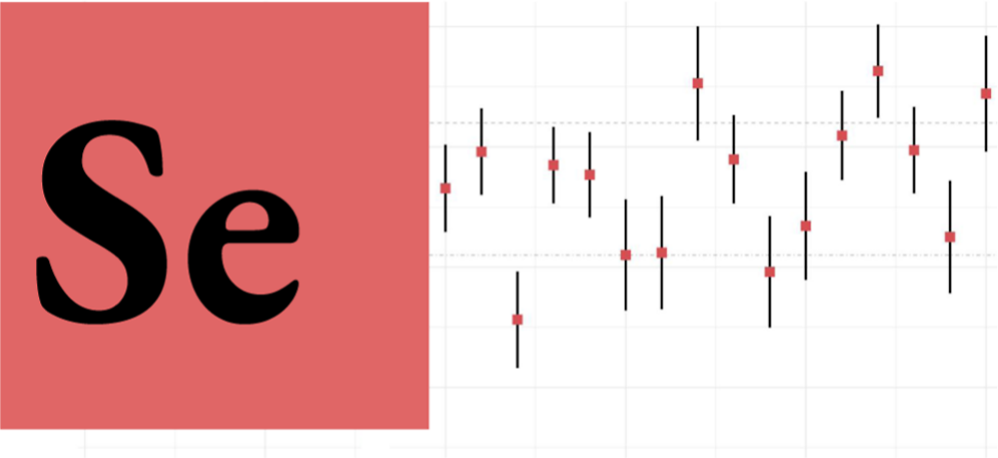

This study introduces a simplified purification method for analyzing ^82^Se/^78^Se isotope ratios in diverse natural samples using hydride generation MC-ICP-MS. Unlike the thiol resin method, which is time-consuming and sensitive to the concentrations of reagents used at individual stages, our proposed alternative is quicker, simpler, and robust. The procedure involves coprecipitation of selenium with iron hydroxide and dissolution in hydrochloric acid. Combining hydride generation and a second cleanup stage achieves sufficient purification for Se isotope ratio measurements. The method is efficient, taking 3–4 h after sample decomposition, utilizing common reagents [HCl, Fe(NO_3_)_3_, NH_4_Cl] without evaporation or clean lab conditions. Results on ^82^Se/^78^Se isotope ratios in various matrices are presented, comparing them with literature data. All isotopic results have been subjected to a newly proposed state-of-the-art approach to uncertainty estimation dedicated to isotope ratio measurements. The approach is based on applying Monte Carlo simulations with consideration of different samples’ results normalized by the expected value. By doing that, we obtained estimated uncertainty for any Se sample with the influence of particular measurements on the final estimation included. We employ a Monte Carlo simulation-based uncertainty estimation approach for isotope ratio measurements, providing estimated uncertainty for each selenium sample.

## Introduction

Selenium (Se) is an essential trace element for many organisms, playing a direct role in promoting healthy growth and maintaining normal physiological functions. Notably, selenium exhibits both poisonous and beneficial influences, with a narrow range between them.^1^ Recently, Se has gained attention also in the fields of geoscience,^2^ environmental science,^3^ paleontology,^4^ botany,^5^ zoology,^6^ microbiology,^7,8^ medicine,^9^ animal husbandry,^10,11^ food, and nutrient sciences.^12^

Selenium has six natural isotopes of masses 74, 76, 77, 78, 80, and 82 with mole fractions in naturally occurring samples of 0.889, 9.366, 7.635, 23.772, 49.607, and 8.731%, respectively. For a long time, thermal ionization mass spectrometry (TIMS) was considered a gold standard for highly precise measurements of Se isotope ratios, particularly in the exploration of natural fractionation of Se isotopes. This method was endorsed by the International Union of Pure and Applied Chemistry (IUPAC) for determining Se isotopic abundances.^13^ Nowadays, most investigations of Se fractionation in natural objects are carried out by using multiple collector inductively coupled plasma mass spectrometry (MC-ICP-MS), which offers better precision and higher sensitivity in comparison with TIMS, especially for elements with relatively high ionization potential.^14^ That is why MC-ICP-MS has found numerous applications for various samples, including yeasts,^15^ chondrites,^16^ seawater,^17^ urban top soils,^3^ and different geological samples.^4,18^

A critical problem in the measurement of isotope ratios using the MC-ICP-MS technique is the purification of the sample. Prior to undertaking isotopic analysis by MC-ICP-MS, it is mandatory to separate the analyte from the sample matrix components that can affect the mass bias on the instrument and can form complex compounds, thereby causing an interference with the element of interest.^19^ It is crucial to ensure unification of the effects occurring during sample introduction (e.g., formation of hydrides). Only then it is possible to properly use the Standard Sample-Bracketing (SSB) method to correct isotopic fractionation in the instrument and thus obtain results of sufficient trueness and precision. SSB involves measuring the isotope ratio of an analyte alternately in its isotopic standard and in the sample. This approach enables the calculation of the isotope ratio, corrected for the mass discrimination effect.

The effectiveness of thiol cotton fiber (TFC) for the quantitative adsorption of Se, as well as tellurium (Te) and antimony (Sb), has been demonstrated.^20^ The main benefits of using TCF are complete yields of Se and the removal of matrix.^21^ Unfortunately, this multistep procedure is time-consuming and labor-intensive. In addition, it requires clean laboratory conditions, experience and precise selection of reagent concentrations. Particularly critical is the last step, where complete desorption of Se is achieved through cotton treatment with nitric acid in a boiling-water bath for 20 min. The amount of nitric acid is a crucial parameter, as too little may result in incomplete desorption (posing a risk of isotopic fractionation), while an excess may introduce significant interferences, even with hydride generation, rendering the resulting selenium-containing solution unsuitable for isotopic measurement.

In this work, we proposed a fundamental simplification of the sample preparation procedure for isotope measurements. We proposed the use of a straightforward sample purification method, previously employed for total content analyses,^22^ and also as a preliminary step in the study of the Ge isotope ratio.^23^ We emphasize here that the procedures of sample purification and analytes separation in isotopic analysis must have different characteristics than procedures for quantitative analysis, particularly in avoiding isotopic fractionation. Therefore, the applicability of methods developed for quantitative analysis in isotopic analyses must be confirmed. In the present work, we have demonstrated that in combination with hydride generation, the proposed method is a convenient way to obtain sufficiently pure Se fraction for isotopic measurements, even when very complex samples are concerned.

The hydride generation (HG) reaction is the most commonly used for Se,^3,4,15,24^ providing the best possible sensitivity and effective matrix removal. Detailed comparison of the techniques of introducing Se-containing samples into MC-ICP-MS confirmed that HG allows the best measurement sensitivity and precision of results.^24^ In addition, this method enables effective sample purification, especially from the elements of the first and second groups of the periodic table. The d-block metals also do not affect hydride formation until their concentration in the sample reaches a critical value.^22^ Generating volatile metalloid hydride has long been the most suitable technique for online separation and speciation investigation of ng to pg amounts of Ge, As, Se, Sb, and Sn.^15,21,24^ This procedure involves reducing the element of interest in the solution to its volatile hydride species using a strong reducing agent, such as NaBH_4_, generating hydrogen (in statu nascendi) upon mixing with the acidified sample solution. The separation of the evolved gas and remaining solution is performed using a dedicated HG system. It is worth mentioning that there is a risk of in situ decomposition of generated hydrides of Ge, Se, etc., in the presence of selected transition metals.^24,25^ Instrumental mass bias is generally corrected using either the SSB or the double-spike method. Essential advantages of the use of HG MC-ICP-MS are as follows.

1. Higher sensitivity, lowering the total amount of element required for one analysis in comparison to sample introduction by nebulization.^24,25^
2. Further separation of the analyte from its matrix, removing potential isobaric interferences.^26^

Another vital issue to be addressed in modern isotope measurements is measurement traceability and comparability of results. The comparability of isotope amount ratio measurement results depends on many factors that have to be considered. The results of isotope ratio measurements are often indicated with only measurement precision instead of expanded measurement uncertainties as recommended by the authoritative Guide to the Expression of Uncertainty in Measurement.^27–29^ Uncertainty budget including only the standard deviation (a measure of precision) of a measurement is particularly unfavorable in the case of measurements by MC-ICP-MS technique. These instruments are specially designed to operate with optimal stability. Moreover, they ensure the simultaneous transfer of individual isotope beams through the spectrometer, which will further improve precision. Because of that, the precision of the measurement itself is often very high and does not adequately describe the measurement uncertainty. Therefore, a systematic evaluation of the uncertainty of isotope measurement becomes imperative.

In this paper, we evaluated two approaches for estimating measurement uncertainty of Se isotope ratios using the SSB method. The first one involves a simple and intuitive estimation based on the difference between a measured value and an expected true value. In the second approach, we developed Monte Carlo simulations extending the first one by including the influence of particular measurements’ standard errors on the uncertainty computations. While Monte Carlo simulation has been used to estimate uncertainties, including isotope measurements,^29^ we propose a new use of this tool. This approach is more astute than currently used methods for uncertainty reporting. The estimations we obtained are intended to apply to any selenium ^82/78^Se isotope ratio measurement conducted using the SBB method. Nevertheless, the uncertainty estimation methodology can be adapted for any element, however, computations require a sufficient amount of measurement data for samples with known true delta values.

## Methods

### Reagents and Standards

All reagents were of analytical reagent grade. All samples and standards were diluted with deionized water (Milli-Q Integral 3 Q-POD Water Purification System, Merck Millipore, Germany). Selected geological and biological reference materials were analyzed to validate the proposed analytical procedure. These include the United States Geological Survey Reference Materials SGR-1 (oil shale, Green River Formation), SCo-1 (cody shale), MAG-1 (marine mud), European Reference Materials BC210a (selenized wheat flour), and selenium-enriched yeast-certified reference material SELM-1. It should be emphasized that these materials are certified, among others, in terms of elemental composition, but the Se isotopic composition is not among the certified values. However, these materials are relatively well available and with published results of the Se isotopic composition.

Hydrofluoric acid (40%), nitric acid (65%), hydrogen peroxide (30%), hydrochloric acid (37%, all Merck Suprapur, Darmstadt, Germany) and perchloric acid (70%; Chem-Lab NV, Zedelgem, Belgium) were used for dissolution geological and biological SRM. Hydrochloric acid (37%, Merck Suprapur, Darmstadt, Germany), iron(III) nitrate nonahydrate, and ammonium chloride (both Merck, Darmstadt, Germany) were used in the purification procedure. Magnesium chloride (Sigma-Aldrich, Milwaukee, WI, USA), sodium chloride and potassium chloride (Merck Suprapur, Darmstadt, Germany) were used to prepare artificial seawater (ASW). In this work, we chose to refer our isotope results to NIST SRM 3149, which is the most often referred to by other authors and has widespread commercial availability. Consequently, we employed it as the bracketing standard in all presented measurements. The preparation of the NIST SRM 3149 solution involved the use of ICP standards for Na, Ca, Mg, Co, Ni, Zn, Pb, Cu, Tl, Hg, Sr, Rb, U, Ba (1 g/L), with the addition of matrix elements. Sodium borohydride (Sigma, Milwaukee, WI, USA) and sodium hydroxide (50%, Fisher Scientific) were used for Se hydride generation. Solution of NaBH_4_ (1.2%, w/v) in 0.01 M NaOH was freshly prepared on a daily base^26^ by successively dissolving 0.25 g of sodium hydroxide and 6.0 g of sodium borohydride in 500 mL of deionized water.

### Sample Preparation

To confirm the correctness of the proposed procedure as a relatively simple and convenient way of Se separation for the isotopic measurements different types of SRMs were analyzed: NIST SRM 3149 with the addition of a matrix (Table 1), artificial seawater, and NASS-4 standard seawater (natural Se content lower than 0.05 μg/L) spiked with NIST SRM 3149, SELM-1 and BC210a. In addition, the following geological standards were analyzed (as samples with low selenium content and a complex matrix): SGR-1, SCo-1 and MAG-1.

**Table 1:** Composition of the NIST SRM 3149 at Se Concentration 0.4 mg/L with the Addition of a Matrix (mg/L)

component	sample A	sample B
Na, Ca, Mg	10	100
Co, Ni, Zn, Pb, Cu	2	20
Tl, Hg, Sr, Rb, U, Ba	1	10

### Enriched Model Samples

To validate the efficiency of the separation procedure, two aliquots of NIST SRM 3149 with two different matrix levels were prepared by spiking NIST SRM 3149 with monoelemental ICP standards (Table 1).

The artificial seawater was prepared by an appropriate dilution of chloride salts of sodium, magnesium, and potassium with Milli-Q water. The solution obtained had concentrations of components, as listed in Table 2. This artificial seawater was spiked with NIST SRM 3149 to a total concentration of Se 0.025 and 0.15 mg/L. All solutions were acidified with concentrated hydrochloric acid to a final HCl concentration of 0.5 M.

**Table 2:** Composition of the ASW

	Na^+^	Mg^2+^	K^+^	Cl^–^
g/kg	10.8	1.3	0.4	18.9

### Geological Standards

To decompose the geological sample, 0.5 g of SGR-1 was weighed in a PTFE vessel, and then 5 mL of nitric acid and 5 mL of perchloric acid were added. The solution was evaporated to 3–4 mL on a hot plate (DigiPREP Jr., Baie D’Urfe, Quebec, Canada) at 80 °C. Then, 5 mL of hydrofluoric acid was added to the residue and evaporated nearly to dryness. That step was repeated 4–5 times to purify the sample more effectively by removing SiF_4_. After decomposition, the residue was dissolved in 7.5 mL hydrochloric acid and heated on a hot plate at 50 °C for 1 h. The final solution, after heating, was transferred into a test tube and diluted with Milli-Q water to 20 mL. To decompose SCo-1 and MAG-1, 0.2 g was weighted in a PTFE vessel of the microwave digestion system. Due to the low concentration of Se in the samples, each was performed in 14 replications, which after the acid digestion step were combined to finally obtain two repetitions. In the first step 7 mL of nitric acid and 1 mL of hydrogen peroxide were added. The obtained mixture was heated for 40 min to 230 °C in a closed system (Ethos Up, Milestone, Sorisole, Italy). The temperature was maintained for 30 min. After cooling, in the second step, 3 mL of hydrofluoric acid was added, and digested at the same conditions. After that, the solutions were transferred into six PTFE vessels, and then 1 mL of perchloric acid was added. The solutions were evaporated to 3–4 mL on a hot plate at 80 °C. Then, solutions were combined to obtain two replications and each of the vessels washed three times with 1 mL of nitric acid. Next, 5 mL of hydrofluoric acid and 5 mL of nitric acid were added and evaporated nearly to dryness. That step was repeated 4 times. After decomposition, the residue was dissolved in 7.5 mL hydrochloric acid and heated on a hot plate at 50 °C for 1 h. The final solution, was transferred to a test tube and diluted with Milli-Q water to 20 mL.

### Biological Samples

To decompose biological samples, each 0.13 g of SELM-1 and BC210a was weighted in a PTFE vessel of the microwave digestion system. Next, 4.5 mL of nitric acid and 0.5 mL of hydrogen peroxide were added. The obtained mixture was heated for 25 min to 210 °C in a closed system. The temperature was maintained for 35 min. After cooling, the solution was transferred to a test tube and diluted to 20 mL with water.

After the dissolution procedure, aliquots of all solutions were analyzed by quadrupole ICP-MS (NexION 300D, PerkinElmer, Waltham, MA, USA) for Se contents, and the recovery was evaluated.

### Separation Procedure

Separation of selenium from the matrix was based on coprecipitating with iron(III) hydroxide.^22^ The novelty and fundamental improvement we have proposed is that this procedure is incomparably simpler compared to the currently used thiol resin method. Followed by hydride generation, which plays role of further separation, equally good results can be obtained. In brief, 20 mL of acidic sample (e.g., 10 mL of NIST SRM 3149 with the addition of matrix +10 mL 0.5 M HCl) was transferred into a PTFE vessel and diluted to 30 mL with Milli-Q water. Then 3.6 g of NH_4_Cl and 150 mg of Fe(NO_3_)_3_·9H_2_O was added [important to note that if the iron content in the material is known from previous analyzes and is high enough, the addition of Fe(NO_3_)_3_ is not necessary]. The obtained solution was heated to approximately 50 °C on a hot plate. After that, the solution was neutralized with pellets of sodium hydroxide and finally adjusted to pH of 2.40 with 0.2 M solution of sodium hydroxide. The solution was heated on a hot plate for 2 h. After that, the obtained precipitate was transferred to the test tube and centrifuged (4200 rpm, 5 min). The iron(III) hydroxide containing selenium (coprecipitation) was dissolved in 5 mL 12 M HCl and diluted with water to the final volume of 10 mL.

### HG MC-ICP-MS Analysis

Selenium isotope ratios were measured at the Biological and Chemical Research Centre of the University of Warsaw using the Plasma 3 multicollector ICP mass spectrometer with 16 Faraday cups (Nu Instruments, Wrexham, U.K.). As mentioned earlier, the most abundant selenium isotope ^80^Se is seriously interfered by a dimer of ^40^Ar. To bypass this problem, three other isotopes (^77^Se, ^78^Se, and ^82^Se), all sufficiently abundant and measurable with high precision, were chosen for isotope ratio measurements. The isotope ^76^Se was not measured due to interferences from ^40^Ar^36^Ar and ^76^Ge, and ^74^Se due to possible interference from germanium and low natural abundance. The isotope ^78^Se is also interfered with ^40^Ar^38^Ar, but ^38^Ar is the least abundant Ar isotope (0.06%). Correcting of Ar interferences is explained below. Three of the Faraday collectors, H8 (^82^Se), Ax (^78^Se) and L2 (^77^Se), were set to register the signals and mass separation of 0.5 atomic mass unit was applied. This configuration was chosen as giving the results of the best trueness. The amplifier boards of the collectors were calibrated every 2 days using an internal 35 V reference signal. Fine-tuning of the MC-ICP-MS instrument was performed before each session. All data sets reported in this paper were collected between April and July 2023.

The HGX-200 advanced membrane hydride generation system (CETAC Technologies, Omaha, NE, USA) was applied for sample introduction to MC instrument. The instrument was running in dry plasma mode. During the development of the method, the Time-Resolved Analysis (TRA) mode and the isotope ratio mode were compared. In the TRA mode, the blank is recorded by measuring the on-peak intensity while introducing only the reagents necessary for the generation of hydrides. In this way, the effect of argon dimers and krypton (plasma gas component) on the Se isotope ratio measurement was corrected. In the isotope ratio mode, we compared blank corrections by electrostatic analyzer deflection (ESA deflection) and between peak zero measurements (both ±0.5 atomic mass units). The results with the best trueness were obtained in the TRA mode and the isotope ratio mode with correction by ESA deflection. In the case of selenium, we do not recommend correcting the blank by measuring the intensity between the peaks.

TRA mode was used for gathering experimental data as it proved to be a better measurement approach when considering transient signals.^30,31^ TRA also enables individual real-blank correction of each registered signal, which allows for the elimination of Ar-derived molecular interferences (like ^40^Ar^38^Ar impact on ^78^Se). Operating parameters for the HG MC-ICP-MS system are listed in Table 3.

**Table 3:** HG MC-ICP-MS Operating Parameters

MC-ICP-MS Parameters	
RF power	1300 W
coolant flow (Ar)	13 L/min
auxiliary flow (Ar)	1.0 L/min
nebulizer gas flow (Ar)	0.64 L/min
interface cones	nickel
Measurement Parameters	
resolution	∼300
cup configuration	H8 (^82^Se), Ax (^78^Se), L2 (^77^Se) and mask on H4
integration time	1 s
measurement mode	time-resolved analysis
blank correction	on-peak blank measurement
replicates	>150
Hydride Generation	
acid reagent	1 M HCl
reducing reagent	NaBH_4_ (1%) mixed with NaOH (0.01 M)
reagents and sample flow rate	0.83 mL/min
sample gas rotameter position	around 15 mm
additional gas flow rate	0.64 L/min

**Figure 1:**
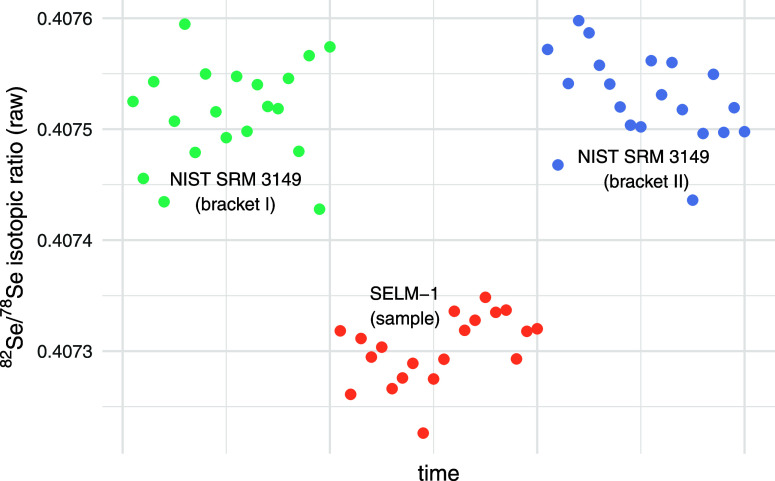
An example of a single measurement of SELM-1 sample with use of the SSB method.

The selenium isotope ratios were determined using the SSB method by sequential measurements of the standard-sample-standard. Such external calibration with NIST SRM 3149 provides the delta value, calculated according following equation
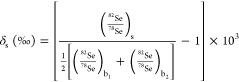
1where s index stands for sample isotope ratio, and b_1_, b_2_ indices for first and second bracketing standards, respectively. Examples of single SSB measurement and subsequent measurements can be found in Figure 1. Since we present only the ^82/78^Se ratios in this work, the results are expressed as δ, leaving a lower index for a specific Se sample if needed. We decided to show ^82/78^Se ratios due to its common presentation in the literature facilitating a more comprehensive comparison of results. At the same time, we want to ensure that the reader is manageable with a multitude of data for two isotope pairs.

Separation of selenium from the matrix by one-stage coprecipitation with Fe(OH)_3_ and then the introduction of selenium into the measurement system in the form of a volatile hydride allowed for obtaining accurate isotopic results for samples with a diverse matrix, including geological samples with Se content as low as 0.89 mg/kg (SCo-1). The minimum weight of such a sample needed for three replicates is 1.5 g, corresponding to 1335 ng Se. The sensitivity we achieved was >1 V ^78^Se per 100 μg/L. All samples were diluted to total Se content around 100 μg/L, except for the NASS-4 sample which was spiked at level 50 μg/L.

### Uncertainty Computation Methodology

The primary objective of this section is to calculate the anticipated level of uncertainty. In other words, we aim to determine a δ value for which we can be 95% confident that it represents the greatest possible difference between a δ value obtained from a new measurement and the expected δ value of the sample. By studying multiple Se samples together, we performed calculations that can be used for any Se sample.

First, we investigate the distribution of measured δ values relative to the true δ value. Let δ_*j*_^*i*^ be δ value of *i*-th measurement of *j*-th sample, and δ_*j*_^true^ the true reference value for the *j*-th sample. This way, we can define the deviation of measurement from the true value as
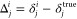
2

We applied the Shapiro–Wilk test to verify whether Δ considered as a random variable, is normally distributed (, *H*_1_: otherwise). Moreover, we used the one-sample Student’s *t*-test to verify if the distribution is concentrated around zero (*H*_0_/Δ^avg^ = 0, *H*_1_/Δ^avg^ ≠ 0). On the complete data set i.e. consisting of all samples we measured, we obtained *p*-values equal to 0.9566 and 0.3578, respectively; detailed *p*-values for individual samples can be found in Table 4. Verifying both hypotheses was essential since, from now on, we can assume that Δ*i*s normally distributed with average value equal to zero.

**Table 4:** *p*-Values of One-Sample Student’s *t*-Test and Shapiro–Wilk Test for Particular Samples and Jointly^a^

	Student’s *t*-test *p*-value	Shapiro–Wilk test *p*-value
NIST	0.7558	0.5950
ASW	0.0371	0.1960
NASS-4	0.5207	0.4153
SELM-1	0.0701	0.9942
BC210a	1.0000	0.5675
SGR-1	0.0052	0.4166
SCo-1	0.3296	0.7336
MAG-1	0.6209	0.5174
jointly	0.3578	0.9566

a ASW stands for artificial seawater, and NIST stands for NIST SRM 3149. Low *t*-test *p*-values in the case of ASW and SGR-1 may mean that either δ_*j*_^true^ value we refer to, or the results of our measurements could be of poor trueness.

Then, we were able to compute the standard deviation of Δ, denoted by SD_Δ_. Again, computations were done for every sample individually, as well as for the complete data set. Note that in cases of individual samples, the standard deviation computation is independent of δ_*j*_^true^ used but it is essential in joint analyses. Results can be found in Table 7.

Finally, we took the 0.975 quantile of a normal distribution with an expected value equal to zero (what justified by the Student’s *t*-test with *p*-value = 0.3578) and standard deviation equal to SD_Δ_ = 0.0701. It enabled us to obtain 95% confidence bound for any δ of newly measured ^82/78^Se observation, i.e.
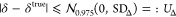
3where . More precise quantiles for specific samples can be found in Table 7.

Now, we would like to note that particular δ values possesses its own standard deviation that we do not considered yet. Let us denote isotope ratios of sample and bracketing standards by μ_s_, μ_b_1__, μ_b_2__ and its standard errors by σ_a_, σ_b_1__, σ_b_2__, respectively (i.e., e.g. values corresponding to three-point clouds in Figure 1). To compute how measurements’ standard errors propagate into standard errors of δ values (denoted by SD_δ_), we can use Taylor series approximation^33^ or differentiation of eq 1.^34^ Both approaches lead us to the same formulation of the propagated standard error of a given δ expressed as
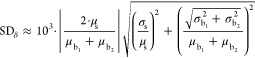
4

**Figure 2:**
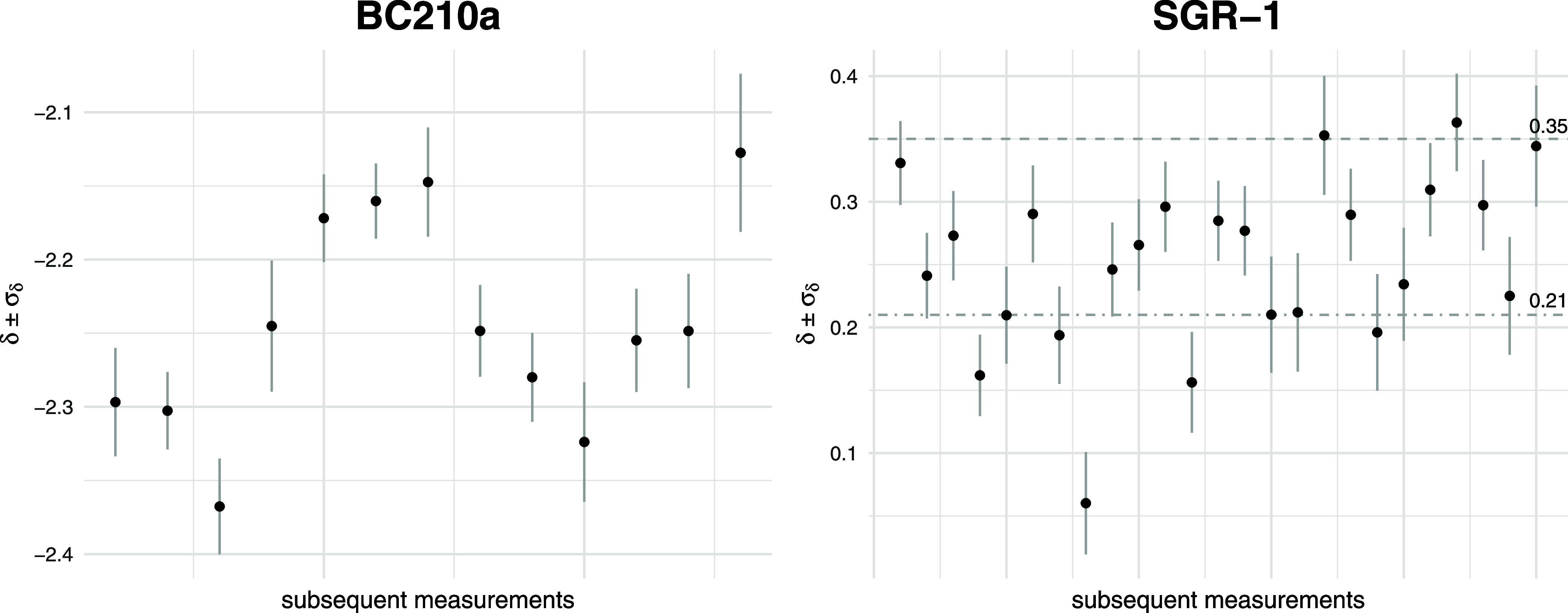
Exemplary sequences of measured δ values with corresponding propagated standard errors SD_δ_ for BC210a and SGR-1 samples. Two SGR-1 reference δ values 0.21^32^ and 0.35^21^ are marked by dashed lines.

Two exemplary courses of experiments for BC210a and SGR-1 samples with annotated propagated errors SD_δ_ for particular measurements can be seen in Figure 2.

So far, all described computations do not differ from widely used methods. Note, however, that in the *U*_Δ_ computations, we have not considered the impact of the standard errors of individual measurements SD_δ_. We postulate that omitting consideration of SD_δ_ may lead to underestimating the uncertainty. Therefore, additional error propagation computations should be conducted. However, since every measured δ possesses its own standard error, obtaining an analytical formula describing how the error propagates is very challenging.

Fortunately, possessing SD_δ_ standard errors enables us to perform Monte Carlo simulations that, in each run, include a random influence of the standard error. It allows us to estimate the uncertainty for a given sample that includes SD_δ_ influence. Each run, we disturbed computation of Δ modifying eq 3 by adding noise from a given δ_s_′ propagated standard error, i.e.
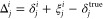
5where . Since every run, we sampled a new error  for every observation separately, we got a different disturbed data set every time. Afterward, on each data set, we repeated SD_Δ_ computations. We repeated the calculations 10^6^ times. Ultimately, we took an average standard deviation over all Monte Carlo runs denoted by SD_Δ_^MC^.

It occurred, the influence of propagated standard error of particular samples on computing expected uncertainty is significant. The Monte Carlo approach resulted in SD_Δ_^MC^ = 0.0963 versus SD_Δ_ = 0.0701 on original data. Again, detailed results can be found in Table 7.

## Results and Discussion

### Hydride Generation Validation

The first step toward implementing the task was optimizing the hydride generation parameters to obtain the maximum sensitivity and the best signal stability. The main parameters subjected to optimization were the gas flow values, the pH of the sample, and finally, the concentration of hydrochloric acid. From the proposed method’s perspective, the influence of HCl concentration on the measurement result is very important. We checked the signal intensity of the sample in acetate buffer (pH 4.8) and then in HCl with increasing concentration, ranging from 0.1 to 6 M. The acetate buffer sample signal was of low sensitivity and was not used further. To demonstrate the effect of HCl concentration, we prepared a Se solution (NIST 3149) with a Se concentration of 200 μg/L and an HCl concentration of 1 M. This solution served as a standard in the SSB method. As samples, we used analogous solutions with a variable concentration of HCl. During the measurements, we intended to check whether, regardless of the concentration of HCl, a δ equal to zero could be obtained. We found no significant effect of the acid concentration on the trueness and precision of the results. However, we observed that a high concentration of HCl resulted in decreased sensitivity. Results are summarized in Table 5.

**Table 5:** Influence of Hydrochloric Acid Concentration on the Trueness and Sensitivity (Relative to 1 M HCl) of the Measurement

sample	δ (‰)	sensitivity (%)
acetic buffer pH ∼ 4.8	N/A	20
0.5 M HCl	–0.03	98
1.0 M HCl	0.07	100
2.0 M HCl	0.04	98
3.0 M HCl	–0.03	90
6.0 M HCl	0.03	80

Then, we tested the effect of iron concentration on trueness and sensitivity. For this purpose, we used a Se solution (NIST 3149) without the addition of Fe as a standard along with analogous solutions with increasing iron concentration (in the range from 10 to 8000 mg/L) as samples. Higher Fe concentrations than 8000 mg/L were not checked as this iron concentration is already well above that needed to carry out the precipitation. Elevated Fe concentrations (above 3000 mg/L) caused a slight decrease in sensitivity but did not adversely affect the trueness of the result. The results of this experiment are summarized in Table 6. We emphasize that in the samples presented in the Table 6, iron was additionally introduced. Its concentration is neither the result of coprecipitation, nor the original iron content of the sample. The next step to validate the method was to subject the standard solution (NIST 3149) to the entire sample preparation procedure. To this end, the solution was subjected to microwave-assisted decomposition in a closed system. Then the whole procedure, including coprecipitating with iron(III) hydroxide, was carried out. The sample preparation procedure did not affect the trueness and precision of the results.

**Table 6:** Influence of Iron Concentration on the Trueness and Sensitivity (Relative to No Spike) of the Measurements

sample	δ (‰)	sensitivity (%)
no Fe spike	0.06	100
10 mg/L Fe	–0.01	99
1000 mg/L Fe	0.00	97
3000 mg/L Fe	0.04	95
8000 mg/L Fe	–0.06	88

### Selenium Isotope Measurements in Natural Samples

As a last and the most important step of method validation, natural samples were measured. Se isotope ratios were measured in materials with Se isotope ratio available in the literature, NIST 3149 solutions spiked with matrix, and seawater spiked with Se NIST 3149. We also present results for BC210a (wheat flour reference material), selenized yeast CRM SELM-1, and for three geological standards SGR, SCo-1 and MAG-1. In Table 7, the results for all samples are summarized.

**Table 7:** Statistics for ^82/78^Se Isotope Ratio Measurements, Computations and Monte Carlo Simulations^a^

	δ^true^ (‰)	δ^avg^ (‰)	Δ^avg^ (‰)	SD_Δ_	SD_Δ_^MC^	*U*_Δ_	*U*_Δ_^MC^	*n*
NIST SRM 3149	0.00	–0.00	–0.00	0.07	0.09	0.13	0.17	54
artificial seawater	0.00	0.04	0.04	0.07	0.08	0.13	0.17	15
NASS-4	0.00	0.02	0.02	0.09	0.10	0.17	0.20	7
SELM-1	–0.68,^30^ −0.66^15^	–0.70	–0.02	0.07	0.08	0.14	0.16	53
BC210a	n.d.	–2.24	0.00	0.07	0.08	0.14	0.16	13
SGR-1	0.21,^32^ 0.35^21^	0.25	0.04	0.07	0.08	0.14	0.16	25
SCo-1	0.175(21)	0.19	0.01	0.04	0.15	0.07	0.29	9
MAG-1	0.21(21)	0.22	0.01	0.04	0.16	0.08	0.32	9
jointly			0.00	0.07	0.10	0.14	0.19	185

a and  are 0.975 quantiles of normal distributions with the given distribution parameters, they should be interpreted as 95% confidence bounds for a difference between measured δ and its true value, as shown in eq 3. Underlined δ^true^ and δ^avg^ (if there were no δ^true^ value we could refer to) were used to compute Δ deviations. Note that for samples considered individually, SD_Δ_ is equivalent to the standard deviation of a given sample. Results for the dataset considered jointly are intended to be estimators for any newly measured selenium sample for which δ^true^ is unknown.

### Uncertainty Evaluation

At this point, we want to explain the values in Table 7 to make it easier for the reader to interpret the data shown therein. δ^true^ means the literature value we have decided to refer to. It is given along with the reference. δ^avg^ is the average δ value of n measurements taken for a given sample. Δ^avg^ is the difference between the average δ value (δ^avg^) from our n measurements and the literature value (δ^true^). In this manner, we introduce a normalization method to calculate combined (joint) expanded uncertainty, serving as a numerical indicator of the trueness of the results. SD_Δ_ is the standard deviation calculated for all n Δ values, i.e. for all n differences between our single result and the literature value. We remind that the SD_Δ_ calculated in this way does not take into account the precision with which individual absolute isotope ratios are measured, i.e. the ratio of ^82/78^Se in the first bracket, in the sample and in the second bracket. Carrying out the propagation of this error and approximating its value using the Monte Carlo method leads to the SD _Δ_^MC^ value. This value differs from the SD_Δ_ value in that it takes into account the precision with which the individual isotope ratios used to calculate each of the n δ values were measured. *U*_Δ_ and *U*_Δ_^MC^ are 95% confidence expanded uncertainty values for SD_Δ_ and SD_Δ_^MC^, respectively. All the results presented in Table 7 were obtained under reproducibility^27^ conditions (different days, conditions of use and different observer).

It should be noted that this elucidation of uncertainty allows us to clearly distinguish which of the components contributes most to the final uncertainty. Suppose the SD_Δ_ and SD_Δ_^MC^ values are similar. In that case, the absolute isotope ratios used to calculate the single δ value have been measured with very good precision. The SD_Δ_ or *U*_Δ_ value mainly results from a significant difference between the single δ values (poor repeatability or reproducibility). If, on the other hand, we observe a large difference between SD_Δ_ and SD_Δ_^MC^ (or *U*_Δ_ and *U*_Δ_^MC^), it indicates that the absolute isotope ratios used to calculate the individual δ values were measured with relatively low precision. This difference can be observed by comparing the results, for example, for BC210a and SCo-1 samples. Poor trueness would be indicated by both high Δ and low *p*-value in the Student’s *t*-test. Therefore, presenting the measurement uncertainty as 2SD may expose us to a fundamental underestimation of the uncertainty value. Suppose we specify the SD of the absolute isotope ratio of the sample (without carrying out error propagation) as our measurement uncertainty. In that case, we will not take into account the component related to the repeatability (or reproducibility) of the method and the trueness of the result. If we present the SD calculated from successive δ values, we consider only the repeatability without taking into account the uncertainties with which individual δ values were determined (and also without taking into account their trueness).

Moving on to the discussion of the results contained in Table 7, first, all the results confirm that purification of a sample by coprecipitation causes no Se fractionation, as δ values for NIST SRM 3149 solutions remain zero after such sample treatment. Moreover, the presence of the matrix elements at concentrations listed in Tables 1 and 2 affects neither the correctness of the measured Se isotopic composition nor the uncertainty values. NASS-4 water spiked with NIST 3149 at the level of 50 μg/L also gave δ satisfyingly close to zero. These three results prove that the Se isotopic composition of seawater samples (as long as the level of Se is high enough) can be successfully measured by the proposed method. In addition to this, we obtained Se isotopic results for biological samples: selenized yeast CRM SELM-1 and wheat flour CRM BCA210a. The result obtained for the SELM-1 is consistent with the literature values, and U values are not higher than for the NIST 3149. It means the sample has been satisfactorily purified, and no matrix effects are observed. The biggest challenge was measuring the isotope ratio in some geological samples, especially silicates. These samples have a very complex matrix and a relatively low Se content. The decomposition of such samples (as described in Sample Preparation) is long and tedious. We selected three geological samples with different matrices: SCo-1 is shale, SGR-1 is oil shale, and MAG-1 is marine mud. We obtained satisfactory trueness for all these samples (low Δ^avg^ value).

It should be noted, however, that the assignment of the expected value for these samples was somewhat cumbersome. For the MAG-1 and SCo-1 materials, we took the expected value reported by Rouxel^21^ (for both, we used the average value of two repetitions shown by the author). For the SGR-1 material, we refer to results presented by Stüeken^32^ and Rouxel.^21^ To recalculate the Se isotopic results presented against the Se ICP Merck standard to results against the NIST 3149, we used the data presented by Carignan.^35^ All samples were analyzed after dilution to a total Se content of 100 μg/L. The exception is the NASS-4 sample, spiked at 50 μg/L. We chose to work with samples at Se content of 100 μg/L because it allowed us to get the maximum number of replicates and thus better estimate the uncertainty.

Moreover, we aimed to demonstrate that satisfactory results can be achieved with minimal amounts of selenium. In the case of geological samples, probably due to the matrix effects, the results were of significantly worse precision and trueness when diluted to about 50 μg/L. For samples SCo-1 and MAG-1 (these two are samples with the lowest Se content, around 1 mg/kg of solid sample), we observed lower precision in measuring absolute ^82/78^Se values also at Se level of 100 μg/L. In the case of samples with a complex matrix and a low selenium content, it might be advantageous to consider introducing a sample solution with a higher Se concentration. However, achieving this would require decomposing a considerably larger sample amount, which poses challenges for technical reasons. In the case of biological and water samples, measuring at Se level of 50–100 μg/L with satisfactory uncertainty was possible.

All acquired data and code used for computations in the R programming language are freely available via our GitHub page https://github.com/PiotrRadzinski/se_purification/.

## Conclusions

In this study, we demonstrated that coprecipitation of selenium with iron(III) hydroxide, which has been used in quantitative analysis with spectrometric detection, can be successfully used as a stand-alone, one-step method of sample purification for Se isotope analysis. The process involves the addition of iron salt, followed by heating and pH adjustment, leading to the precipitation of iron hydroxide along with the coprecipitation of selenium from the sample. Subsequent dissolution of the precipitate in concentrated acid completes the procedure. In light of the above results, such a simplified process is sufficient to obtain correct isotopic results, particularly when coupled with hydride generation (for three repetitions 0.9 μg Se is required). Furthermore, we demonstrated that the parameters that are relatively difficult to control during sample preparation using this method (iron concentration and HCl concentration) do not affect the trueness of the results significantly.

Consequently, this means that the isotopic result is not very dependent on these two parameters, eliminating the need for meticulous monitoring. After such one-step purification and generation of volatile hydrides, the analytical signal is of such good quality that it is possible to measure the relative isotopic fractionation (δ values) by the simplest possible method (SSB) with no need for additional corrections. The commonly used selenium separation using ion-exchange resin is very time-consuming and sensitive to the reagents’ concentrations. The presented method is much simpler, shorter and incomparably more robust to the variability of experimental conditions.

Similarly to the method with ion-exchange resin, the presented method allows to concentrate Se by changing the ratio of the volume of the sample and the volume of acid in which the precipitate of iron hydroxide is dissolved. We emphasize that the presented method does not eliminate the necessity of complete digestion of the sample. In our opinion, this process remains a critical step, most susceptible to poor execution, selenium losses, contamination, and Se isotopic fractionation especially when complex geological samples are concerned.

An equally important aspect of this work is the development of the method of estimating the measurement uncertainty. The presented approach allowed us to determine a realistic *U* value for which we can be 95% confident that it represents the most probable difference between a δ value obtained from a new measurement and the expected δ value of the sample. Propagation of error and approximating its value using the Monte Carlo method allowed us to take into account the influence of measurement errors of individual absolute ^82/78^Se values on the final uncertainty of determining the δ value. Note that this is an uncertainty of isotope ratio, not selenium concentration. The proposed approach also considers the effect of conducting measurements in repeatability and reproducibility conditions and enables the detection of systematic error (Δ and *p*-value in Student’s *t*-test). We have shown and discussed how various factors can affect the value of uncertainty and proposed an approximate value of uncertainty (jointly in Table 7) that can successfully describe the uncertainty of the measurement of the ^82/78^Se isotope ratio carried out using the SSB HG MC-ICP-MS measurement system for samples with a diverse matrix. At the same time, the proposed mathematical approach can be used more universally for isotope measurements of any isotopic pair and with sample introduction in any way, as long as the SSB measurement regime is maintained.
